# Field Emission Current Stability and Noise Generation Mechanism of Large Aspect Ratio Diamond Nanowires

**DOI:** 10.3390/s25092925

**Published:** 2025-05-06

**Authors:** Yang Wang, Jinwen Zhang

**Affiliations:** School of Integrated Circuits, Peking University, Beijing 100871, China

**Keywords:** diamond nanowires, field electron emission, current stability, surface work function, noise power spectrum

## Abstract

This paper reports the field emission (FE) current stability of a diamond nanowire (DNW) array. Assembled with a silicon anode with a 1.03 μm gap, the FE properties, as well as the current stability of the DNW cathode, were systematically evaluated in a vacuum test system under different vacuum degrees, current densities, and atmospheres. Experiments demonstrate that lower pressure and current density can improve FE properties and current stability. In addition, compared to air and compressed air, DNWs exhibit higher FE properties and current stability in N_2_. DNWs achieve a remarkably low turn-on field of 1.65 V/μm and a high current density of 265.38 mA/cm^2^. Notably, they demonstrate merely 0.70% current fluctuation under test conditions of 1.2 × 10^−4^ Pa and 0.1 mA/cm^2^. Additionally, based on the Fowler–Nordheim theory, the change in work function after gas adsorption was analyzed, and the noise generation mechanism was derived from the noise power spectrum. The current exponent is determined as 1.94, while the frequency exponent ranges from 0.92 to 1.32, confirming that the dominant noise mechanism in DNWs arises from surface work function fluctuations due to the adsorption and desorption of residual gas.

## 1. Introduction

Field electron emissions enable a stable current at room temperature, characterized by high efficiency, low power consumption, fast response, and easy miniaturization [1]. This technology has found extensive applications in display devices [2], microscopic imaging [3], switching devices [4], and electric thrusters [5]. Hydrogen-terminated diamonds exhibit significant negative electron affinity (NEA), greatly lowering the energy barrier for electron emissions from the material surface. Micro/nano tip arrays with large aspect ratios have been achieved on various diamond substrates to enhance FE performance, including single crystal [6], polycrystalline [7], nanocrystalline (NCD) [8], and ultra-nanocrystalline (UNCD) [9], featuring tip diameters ranging from 24 nm to 1.5 μm and aspect ratios below 10. Notably, the N-UNCD tip array exhibits a low turn-on field of 2.6 V/μm and a current density of 2.38 mA/cm^2^ under an external electric field of 5.8 V/μm [9]. DNWs possess extraordinary mechanical properties, thermal conductivity, and electrical characteristics, which have attracted widespread attention in low-threshold electric field emissions, high-performance nanoelectromechanical switches, and biosensors [10].

Current stability is a critical feasibility criterion for next-generation FE devices, especially for cold cathode materials in electron source applications. A stable emission current with minimal fluctuations has proven to be more essential than a low turn-on field and high current density [11]. The emission stability of FE devices mainly depends on the physical and chemical stability of the cathode material, including resistance to chemical erosion and ion bombardment from residual gases, tolerance to pressure differentials, and environmental stability in different gas environments. Diamond, possessing the strongest covalent bonds, maintains structural stability even under high FE current densities [12], making it an optimal material for FE cathodes with stable current emissions and extended service life. Uppireddi et al. investigated the FE temporal stability of UNCD thin films deposited by hot filament chemical vapor deposition (HFCVD). The emission current of UNCD films grown at 90% Ar exhibits deviations within 15% from average, meeting field emission display (FED) application requirements while demonstrating a high-frequency stability better than 1% [13]. Golubkov et al. prepared a SiC FE array coated with an NCD film. The current fluctuations were reduced to 5% compared to 23% in the uncoated array [14]. Gao et al. fabricated spherical diamond microcrystalline aggregate films using a microwave plasma chemical vapor deposition (MPCVD) system, demonstrating a current fluctuation below 2.6% over 90 min, superior to carbon nanotubes (CNTs) and amorphous carbon (a-C) [15]. Koinkar et al. investigated the FE current spectrum of NCD films synthesized with a 5000 ppm boron concentration, observing “step” and “spike” fluctuations characterized by $1/f^{\alpha }$-type noise. A spectral analysis yielded an α value of approximately 0.8 [16], indicating that the adsorption and desorption processes of residual gases constitute the primary source of the fluctuations in an FE current.

So far, research efforts have predominantly concentrated on current stability evaluations under singular high-vacuum conditions, with a lack of studies on current stability under different testing conditions and mechanistic analysis of current fluctuations. Moreover, current research is largely confined to diamond films, with a conspicuous absence of reports on the current stability of large aspect ratio tip arrays, particularly DNWs. Our research group reported the FE characteristics of DNW arrays prepared by annealing NCD in the air for the first time in the field, demonstrating the feasibility of achieving both a reduced turn-on field and enhanced FE current densities, which effectively reduces the preparation cost and operation difficulty and paves the way for the practical application of large-area diamond field emission arrays [17]. Furthermore, the structure, composition, and FE testing system of the DNW array were thoroughly investigated. Additionally, the FE characteristics of the NCD film and surface hydrogenated DNWs were analyzed and compared [18]. In this paper, the FE properties and current stability of DNWs under different vacuum degrees, current densities, and atmospheres and their mechanism were investigated systematically. The current-voltage (I-V) characteristics and current stability measurements were conducted and comparatively analyzed. Stability parameters were extracted from Fowler–Nordheim (F-N) plots, while fast Fourier transform (FFT) was employed to obtain the FE noise power spectrum to elucidate the factors and mechanisms of the FE properties and current stability.

## 2. Sample Preparation and Characterization

Single-cast n-type<100>Si was immersed in a suspension of 50 nm diameter diamond particles and ultrasonically scraped for more than 1 h. NCD films were grown by a microwave plasma chemical vapor deposition (MPCVD) system, and then the NCD sample was annealed in air for 120 min in a muffle furnace to obtain DNWs. Finally, the sample was hydrogenated in an MPCVD chamber for 20 min. The detailed growth and preparation conditions of the DNWs are thoroughly documented in [17].

**Figure 1:**
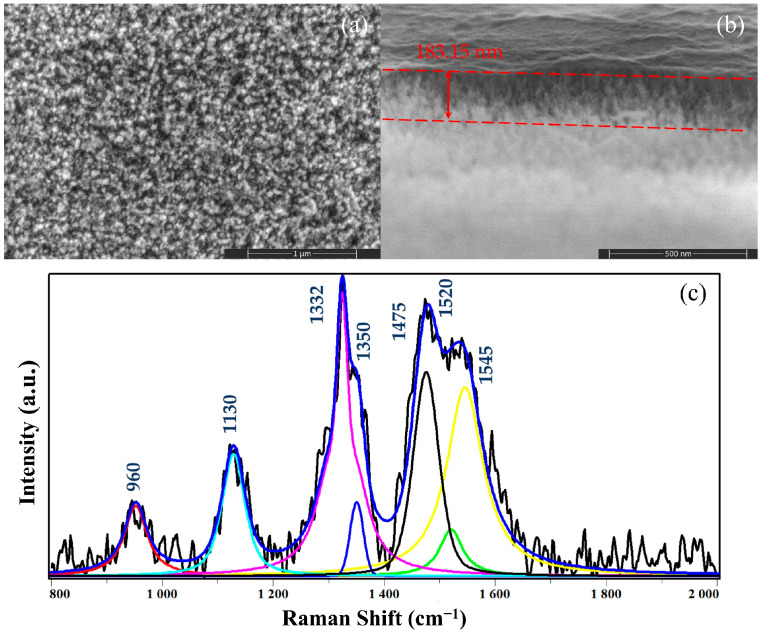
Morphology and composition characterization of the DNWs: (**a**) SEM image of the top surface; (**b**) SEM image of the cross-section; (**c**) Raman spectrum [18] © IOP Publishing. Reproduced with permission. All rights reserved.

The morphology and composition characterization of the DNWs are comprehensively described in [18]. The scanning electron microscope (SEM) images and Raman spectrum of the DNWs are shown in Figure 1. The top surface of the DNWs (Figure 1a) demonstrates the formation of a nanowire array structure with a high distribution density and small curvature radius. The local high-magnification cross-section image (Figure 1b) shows numerous elongated nanowires perpendicular to the substrate direction, demonstrating the formation of the DNWs, with an average length of 183.15 nm, a median diameter of about 9.6 nm, a spacing of about 35.8 nm, an average tip radius of about 3.43 nm, and a density of 1.0 × 10^11^ cm^−2^, yielding an exceptional aspect ratio of approximately 19:1. The composition was characterized using the Raman spectrum at a 532 nm wavelength (Figure 1c), and distinct peaks can be found for the sp^3^ diamond at 1332 cm^−1^ and the D and G bands of the sp^2^ at 1350 cm^−1^ and 1545 cm^−1^, respectively. In addition, the characteristic peaks of trans-polyacetylene at 1130 cm^−1^ and 1475 cm^−1^ are clearly visible, indicating that the sample is NCD.

## 3. FE Properties and Current Stability Test System and Electrical Demonstration

**Figure 2:**
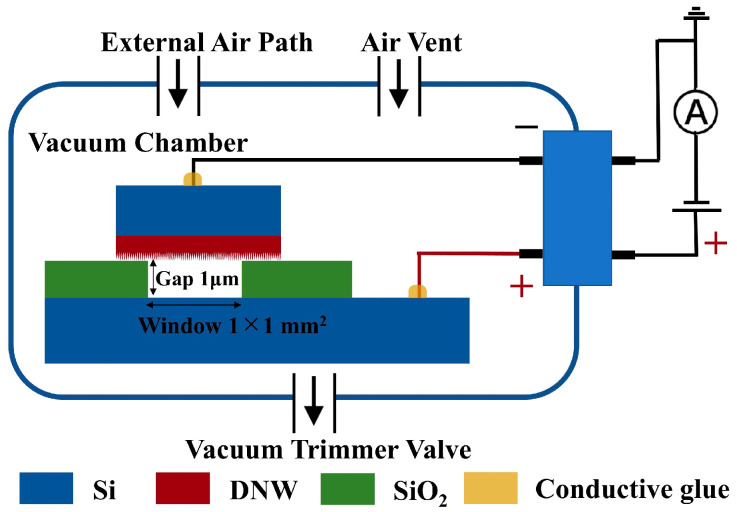
Schematic diagram of the electrical connection inside the vacuum chamber of the flat plate FE test structure.

Firstly, a flat plate FE test structure with a 1.03 μm gap and an anode window area of 1.04 mm^2^ was designed and fabricated. The SiO_2_ layer not only precisely controls the gap but also serves as an electrical isolation between the anode and cathode. Then, the test structure was installed in a vacuum testing system with a vacuum degree up to 10^−4^ Pa. Detailed images of the vacuum test system and electrically connected FE test structure during the sample stage inside a vacuum chamber are available in [18]. The vacuum degree inside the chamber was regulated through a trimmer valve. N_2_ or compressed air can be introduced into the vacuum chamber through an external air path, and air can be introduced through a vent. The schematic diagram of the electrical connections inside the vacuum chamber is shown in Figure 2. Finally, FE properties and current stability tests were conducted under different conditions. FE I-V curves were recorded using the voltage scanning mode of an Agilent 4156B semiconductor parameter analyzer (Agilent Technologies, Santa Clara, California, United States), with a scanning voltage range of 1–100 V. Using the current-time mode, the variation curves of the FE current at a fixed voltage were measured, with one observation point taken every 1 s for 3600 s.

## 4. Results and Discussion of FE Properties and Current Stability

### 4.1. Effect of Vacuum Degree

#### 4.1.1. FE Properties

**Figure 3:**
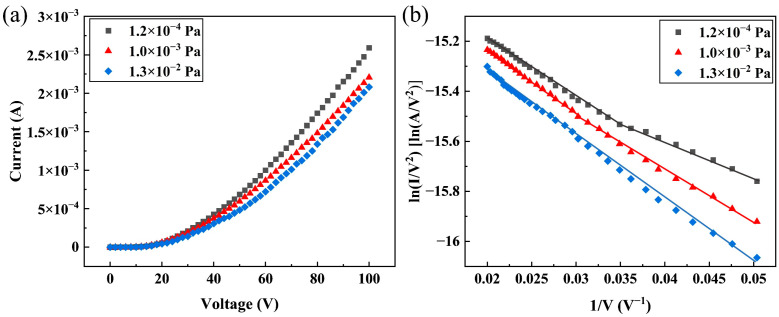
FE properties of the DNWs under different vacuum degrees: (**a**) I-V curves; (**b**) F-N curves and their piecewise linear fitting, where I is the FE current, and V is the applied voltage.

Table 1 summarizes the results of the FE properties of the DNWs in the vacuum range from 1.2 × 10^−4^ to 1.3 × 10^−2^ Pa. The corresponding I-V curves are shown in Figure 3a. Analysis reveals a clear pressure dependence: the turn-on electric field ($E_{t}$) required to generate a current density of 10 μA/cm^2^ increases from 1.84 to 3.50 V/μm, while the maximum FE current density $J_{0}$ at 100 V decreases from 249.04 to 200.08 mA/cm^2^ as pressure rises. The exceptional FE performance of hydrogenated DNWs can be attributed to their nanoscale curvature radius, large aspect ratio, and NEA, as thoroughly characterized in [18].

The variations in FE properties are probably influenced by various factors, including oxidation, gas adsorption, and sputtering-induced damage [19]. Firstly, oxygen-containing substances such as O_2_, H_2_O, and CO_2_ are known to degrade FE performance. However, experimental measurements confirm that it is difficult for DNWs to oxidize rapidly below 600 °C [20]. Secondly, if sputtering causes damage to the tips, which is the reason for the change in FE properties, then the FE degradation would demonstrate irreversible, time-dependent deterioration. However, when the pressure is reduced from 1.3 × 10^−2^ to 1.2 × 10^−4^ Pa, the FE property shows a fairly stable recovery after 20 min. This reversible behavior confirms the structural integrity of the DNW tips before and after FE, proving that it is difficult for oxidation and sputtering damage to affect FE properties during the testing process.

**Figure 4:**
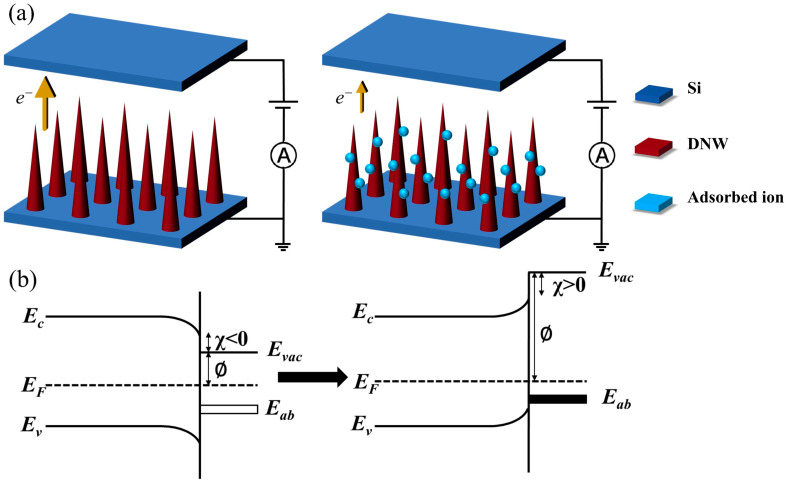
Schematic diagrams of the DNWs before and after gas adsorption: (**a**) process diagram; (**b**) energy band diagram.

Therefore, surface gas adsorption is identified as the predominant factor influencing FE property variations. The schematic diagram of the DNWs before and after gas adsorption is shown in Figure 4. For clean hydrogenated DNWs, due to the lower electronegativity of hydrogen (2.10) compared to carbon (2.55), C(δ^−^)-H(δ^+^) dipoles are formed on the surface, resulting in an NEA and low surface work function. When exposed to gas, first, gas molecules physically adsorb onto the surface of DNWs. These adsorbed species, typically possessing higher electronegativity, extract electrons from the conduction band, inducing upward band bending. This results in a positive electron affinity and an increase in surface work function, making it difficult for electrons to be emitted from DNWs [21], ultimately elevating $E_{t}$ and reducing emission current. Assuming that each adsorbed gas generates a dipole moment $\mu_{0}$ with DNWs, the change in work function caused by gas adsorption is as follows [22]:

(1) $$∆\emptyset =\frac{eN_{0}\mu_{0}}{\varepsilon_{0}}$$

where ∅ is the work function of the FE material, the work function of hydrogenated diamond reported in the literature is 4.9 eV [23], $N_{0}$ is the number of adsorbed gases per unit area, e is the electron charge, and $\varepsilon_{0}$ is the vacuum dielectric constant. ∅ varies with the concentration of the adsorbed gas, which, in turn, is related to the vacuum degree. Therefore, FE properties of the DNWs exhibit significant pressure-dependent behavior.

The FE phenomenon is described by Fowler–Nordheim theory as follows:

(2) $$J_{eff}=\frac{A{E_{l}}^{2}}{\emptyset }exp\left(-\frac{B\emptyset^{1.5}}{E_{l}}\right)$$

where $J_{eff}$ is the effective FE current density, *A* = 1.54 × 10^−6^ (A·eV/V^2^), *B* = 6.83 × 10^7^ (V/((eV)^1.5^)·m), and $\;E_{l}$ is the local electric field at the cathode tip. In [18], we have derived the following relationship between FE current I and applied voltage V from Equation (2):

(3) $$\ln\left(\frac{I}{V^{2}}\right)=-\frac{B\emptyset^{1.5}d}{\beta }\frac{1}{V}+ln\frac{SA\beta^{2}}{\emptyset d^{2}}$$

where I is the field emission current, V is the applied voltage, d is the gap distance between the cathode and anode, β is the field enhancement factor, and S is the effective field emission area. It can be seen that $\ln\left(I/V^{2}\right)$) has a linear relationship with 1/V, with the slope related to the work function ∅ and the field enhancement factor β.

Since DNWs do not undergo any geometric degradation that leads to a permanent decrease in FE current, β remains constant, and the change in the slope of the F-N curve reflects the change in ∅ so that the slope K of $\ln\left(I/V^{2}\right)$ versus the 1/V curve is proportional to $\emptyset^{3/2}$. Therefore, the amount of change in ∅ is the following [24]:

(4) $$\emptyset_{A}/\emptyset_{B}={\left(K_{A}/K_{B}\right)}^{2/3}$$

where $\emptyset_{A}$, $\emptyset_{B}$, $K_{A}$, and $K_{B}$ are ∅ and the slope of the F-N curve before and after the change in the state of the FE cathode, respectively.

The FE F-N curves of the DNWs under different vacuum degrees are shown in Figure 3b. Segmented linearity in F-N curve slopes with applied voltage was observed, which originates from the variation in the number of active emitters under different electric field strengths. At low applied electric fields, only partial emitters reaching the turn-on field emit current, while at high applied electric fields, more of the remaining emitters also participate in field emission, making the field enhancement factor represent the average value of the entire DNW array [18,25]. Through piecewise linear fitting of the F-N curves in Figure 3b and according to Formula (4), we derived the rate of ∆∅ under different vacuum degrees based on slope variations at high electric fields. The fitting results and relative work function changes compared to 1.2 × 10^−4^ Pa are summarized in Table 1. As the pressure increases from 1.2 × 10^−4^ to 1.3 × 10^−2^ Pa, the work function increases by 21.66%.

#### 4.1.2. FE Current Stability

**Figure 5:**
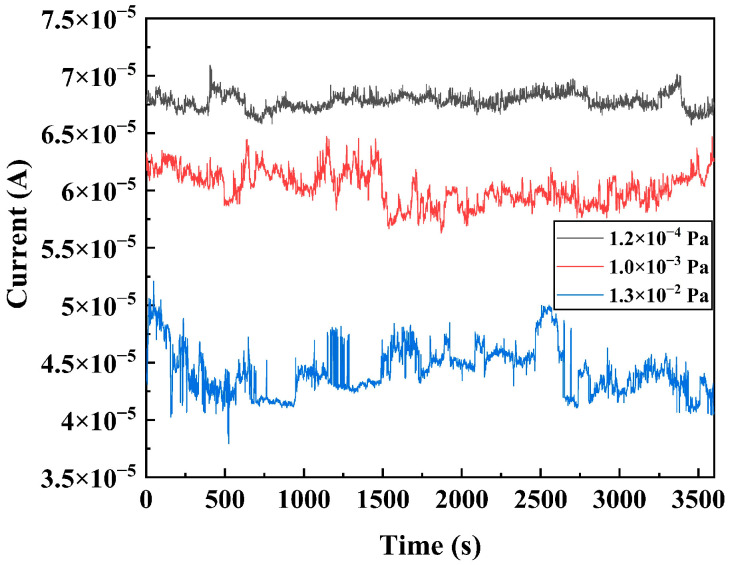
FE current stability curves of the DNWs under different vacuum degrees.

Under a vacuum degree of 1.2 × 10^−4^ to 1.3 × 10^−2^ Pa, a constant voltage of 20 V is applied to measure the stability curve of current over time, as illustrated in Figure 5. The parameters are summarized in Table 2. DNWs exhibit current fluctuations in the form of spikes originating from short-time variations and steps arising from long-time variations. The average value of current $\bar{I}$, standard deviation δ, and fluctuation rate of the stability curve are systematically calculated and presented in Table 1. $\delta /\bar{I}\times 100\%$ is defined as the fluctuation rate of the current, where lower values indicate superior FE stability. As pressure increases from 1.2 × 10^−4^ to 1.3 × 10^−2^ Pa, δ rises from 9.34 × 10^−7^ to 2.11 × 10^−6^ A, accompanied by an increase in the fluctuation rate from 1.37% to 4.76%. The fluctuation of FE current is mainly caused by the change in ∅ due to the adsorption, desorption, and migration of residual gas molecules induced by ion bombardment. Gas pressure determines the number of ions produced by ionization. The number of ions produced per unit of time can be estimated by Formula (5) [26]:

(5) $$N=\frac{\sigma }{ekT}IPd$$

where P is pressure, k is the Boltzmann constant, T is the absolute temperature, and σ is the gas ionization cross-section. When ion bombardment causes desorption of adsorbed molecules, the current exhibits a step-like fluctuation, while when ion bombardment leads to transient migration of adsorbed molecules, the current exhibits a spike-like fluctuation [27]. According to Formula (5), the number of ions produced per unit of time exhibits direct proportionality to P. Consequently, the current fluctuation rate increases with rising pressure.

### 4.2. Effect of Current Density

#### 4.2.1. FE Properties

**Figure 6:**
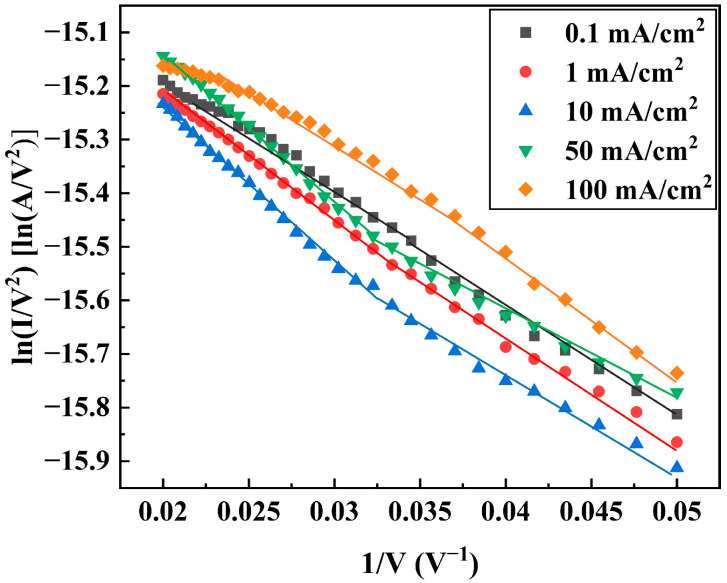
FE F-N curves of the DNWs and piecewise linear fitting results at different current densities, where I is the FE current, and V is the applied voltage.

After stabilizing the DNWs at the corresponding current density for over 4000 s by applying external voltages, a scanning voltage of 0–100 V was applied for field emission testing. The FE properties of the DNWs are summarized in Table 3 for current densities ranging from 0.1 to 100 mA/cm^2^. As the test current increases, the FE properties first decrease and then recover to some extent. The FE F-N curves of the DNWs under different test current densities and their piecewise linear fitting results are shown in Figure 6. The rates of ∆∅ under varying test current densities were determined based on the slope variations in the high applied electric field regions of the F-N curves in Figure 6. The piecewise linear fitting results of F-N curve slopes and the relative work function changes are listed in Table 3. As the test current density increases, ∅ first increases and then decreases.

**Figure 7:**
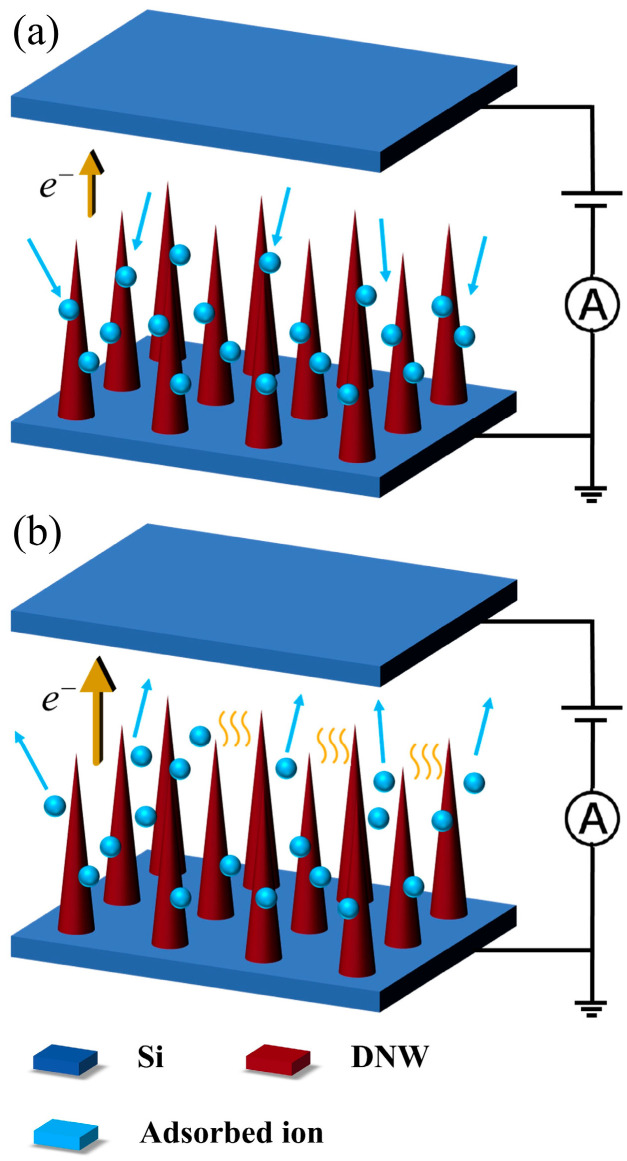
Schematic diagram of the FE states of the DNWs at different current densities: (**a**) field-induced adsorption mode; (**b**) current-induced desorption mode.

Zhao [28] found that the FE properties and change in ∅ of carbon nanomaterial cathodes exhibit a strong dependence on the emission state at different current densities. At low current densities below 10 mA/cm^2^, DNWs operate in field-induced adsorption mode, as shown in Figure 7a. With the increase in current density, the bombardment of emitted electrons is enhanced, causing more gas molecules to desorb from the anode surface. At the same time, the strong local electric field at the tip of the DNWs further attracts adsorbed molecules and enhances the gas adsorption, increasing ∅, resulting in an increase in $E_{t}$ and a decrease in $J_{0}$. However, as the current density further increases, in the high-emission current region, the DNWs transition to a current-induced desorption mode, as shown in Figure 7b. The joule heat of the high FE current causes desorption of the adsorbed gas and displacement from the adsorption site, which gradually reduces ∅, thereby improving FE properties.

#### 4.2.2. FE Current Stability

**Figure 8:**
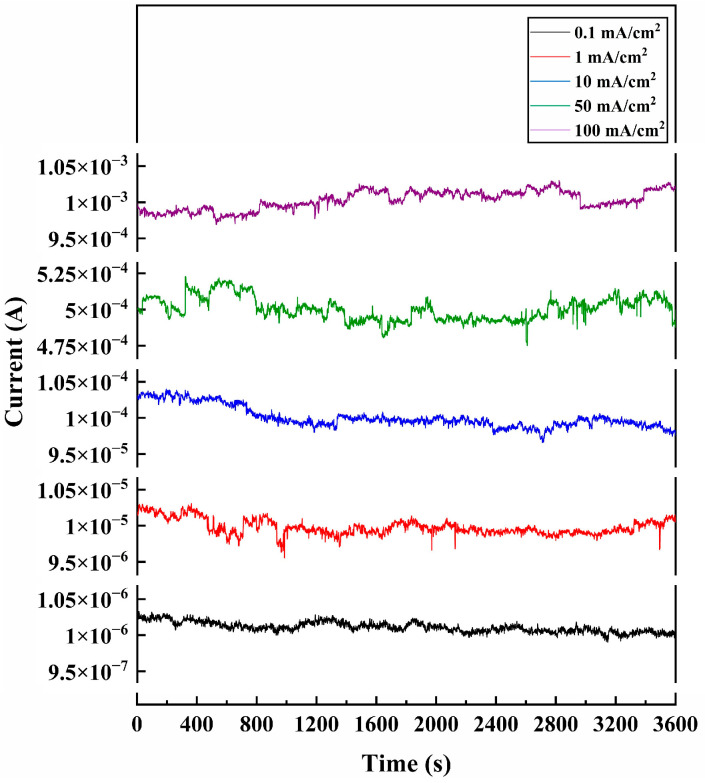
FE current stability curves of the DNWs at different current densities.

The stability curves of the DNWs under different current densities are demonstrated in Figure 8, with corresponding parameters shown in Table 4. The fluctuation rate exhibits a non-monotonic dependence on current density, initially increasing before decreasing at high currents. As the test current density increases, the emission state of the DNWs switches. When the test current density is below 50 mA/cm^2^, according to Formula (5), the number of ions produced per unit time is proportional to the FE current. The increase in current density leads to an increase in the number of ions produced, and enhanced ion bombardment promotes continuous adsorption/desorption and migration of gas molecules on the surface of the DNWs. The current fluctuation rate increases with the rise of current density. As shown in Table 4, the field emission current fluctuation rate increases from 0.70% to 1.61%. When the test current density further rises to 100 mA/cm^2^, excessive joule heat causes a decrease in the number density of adsorbed gases, which reduces the current fluctuation rate, decreasing from 1.61% to 1.30%.

### 4.3. Effect of Atmosphere

#### 4.3.1. FE Properties

**Figure 9:**
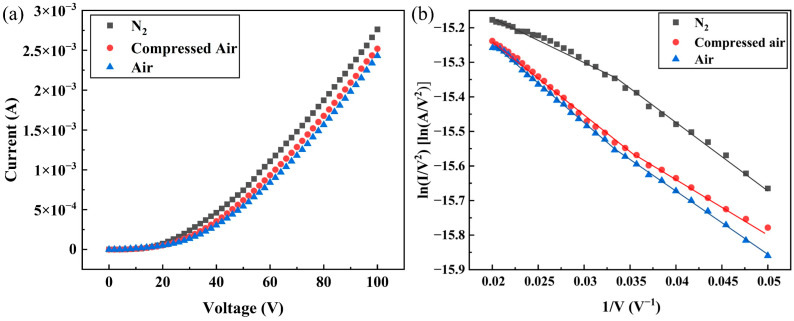
FE properties of the DNWs under different atmospheres: (**a**) I-V curves; (**b**) F-N curves and their piecewise linear fitting, where I is the FE current, and V is the applied voltage.

Ranajoy et al. [29] experimentally confirmed that moisture adsorption in air elevates the work function of silicon field emission tips, causing substantial performance degradation, while field emission arrays in N_2_ show minimal work function variation with only 10% current degradation over 72 h. Filtered and dried compressed air significantly reduces water vapor content while also removing particulates and volatile organic pollutants, making it more suitable than air for standardized testing conditions and working environments for field emission cathodes. Therefore, N_2_, compressed air, and air were selected for comparative experiments. The FE properties of the DNWs under N_2_, compressed air, and air are summarized in Table 5. The I-V curves under different atmospheres are shown in Figure 9a. DNWs reveal optimal FE performance under N_2_, with an $E_{t}$ of 1.65 V/μm, lower than 1.86 V/μm in compressed air and 2.13 V/μm in air. Correspondingly, $J_{0}$ at 100 V reaches 265.38 mA/cm^2^, exceeding 248.08 mA/cm^2^ in compressed air and 233.65 mA/cm^2^ in air. The FE F-N curves of the DNWs under different atmospheres and their piecewise linear fitting results are shown in Figure 9b. The piecewise linear fitting results of F-N curve slopes and the rate of ∆∅ in the high applied electric field regions are summarized in Table 5. Compared with N_2_, ∅ in compressed air increases by 11.45%, and ∅ in air increases by 16.99%.

The adsorption energy and ∅ of different gases adsorbed on the surface of a hydrogen-terminated diamond are shown in Table 6. The adsorption energy $E_{ad}$ is the energy of the surface with the adsorbate relative to their respective energies when isolated from each other. Firstly, the magnitude of $E_{ad}$ indicates the possibility of adsorbed gas adhering to the diamond and its subsequent stability on the surface. In an oxygen-containing atmosphere, O_2_ demonstrates stable adsorption on the DNWs due to the negative value of $E_{ad-O_{2}}$, while N_2_ exhibits negligible adsorption. Secondly, the transfer charge between the DNWs and adsorbed molecules correlates strongly with the electron absorption ability of the adsorbed molecules. As the electronegativity of O (3.44) is higher than that of N (3.04), O_2_ exhibits stronger electron absorption ability than N_2_ molecules, resulting in more significant ∅ elevation than N_2_ adsorption. This fundamental difference explains the superior FE performance in N_2_ versus compressed air. The compressed air used for the test is at a dew point of −35 °C, containing significantly reduced moisture content compared to air conditions. The negative value of $E_{ad-H_{2}O}$ indicates that H_2_O can form stable adsorption, and H_2_O adsorption also increases ∅. In addition, according to Formula (1), the polar nature of H_2_O molecules introduces an additional field-dependent dipole enhancement, where applied electric fields amplify molecular dipoles, causing additional ∅ increases, leading to inferior FE properties in air compared to compressed air.

#### 4.3.2. FE Current Stability

**Figure 10:**
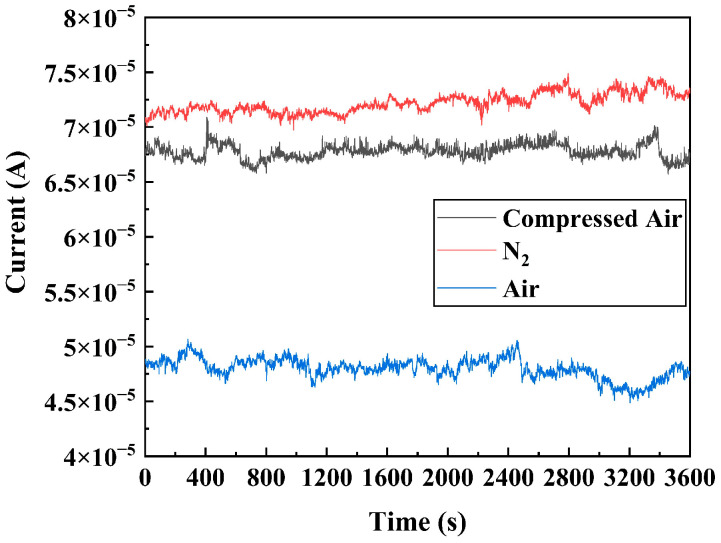
FE current stability curves of the DNWs under different atmospheres.

The stability curves of the DNWs under different atmospheres are shown in Figure 10, with relevant parameters presented in Table 7. The DNWs under N_2_ exhibit the lowest fluctuation rate of 1.25%, while the fluctuation rate in air is the highest, at 1.92%. According to Formula (5), the atmospheric sensitivity in the fluctuation rate is related to the ionization cross-section σ of different gases, which is defined as the probability of ionization collisions of the incident particles as they traverse a gas or a solid. At the same incident electron energy, $\sigma_{H_{2}O}>\sigma_{O_{2}}>\sigma_{N_{2}}$, H_2_O produces the largest number of ions through electron bombardment and generates more current fluctuations than O_2_ and N_2_ at equivalent pressure, thus exhibiting significantly higher fluctuation rates in air.

### 4.4. FE Current Noise Power Spectrum of DNWs

**Figure 11:**
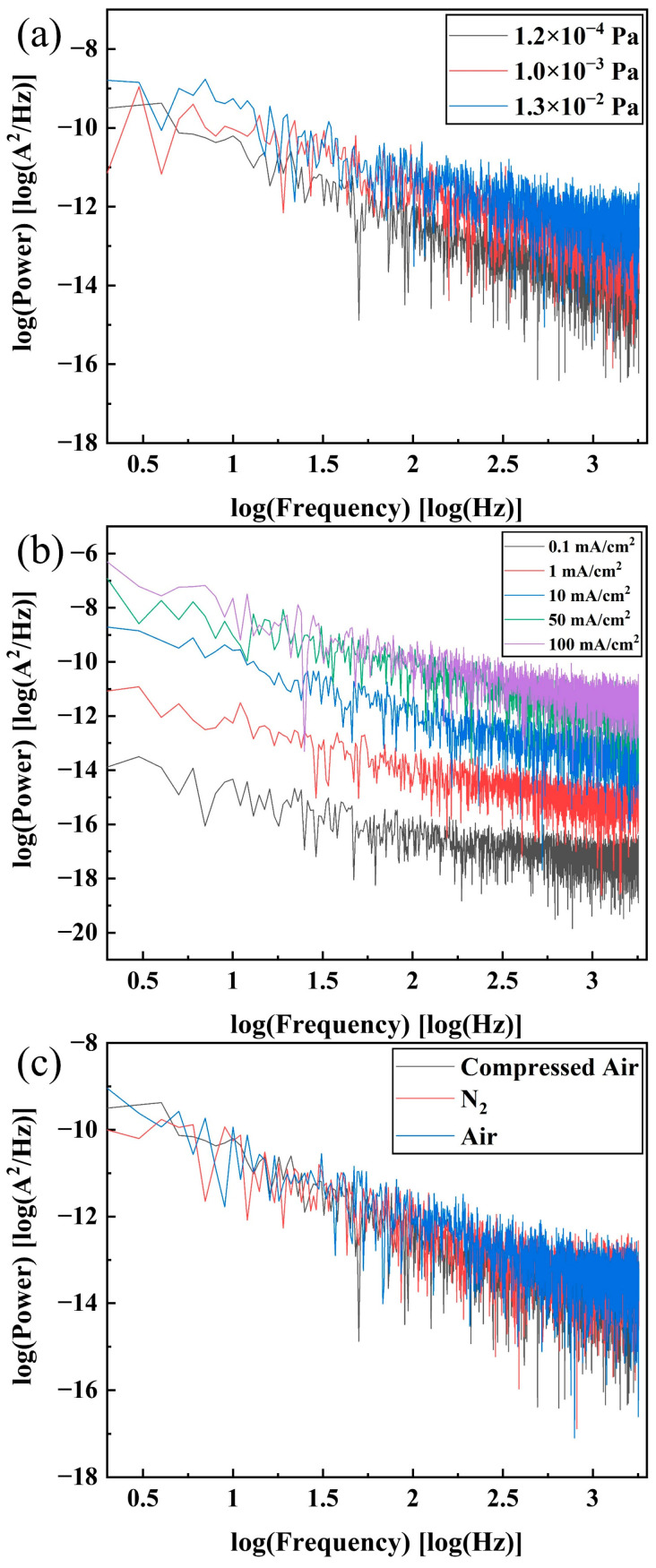
Noise power spectrum of the DNWs under (**a**) different vacuum degrees, (**b**) different current densities, and (**c**) different atmospheres.

FFT was applied to the current stability curves to obtain the current noise power spectrum of the DNWs, as shown in Figure 11. FE noise increases with the rise of pressure and current density. The spectral noise power follows the generalized form $P_{n}\left(f\right)=A_{0}\left(I^{\alpha }/f^{\xi }\right)$, where α and ξ are the current exponent and frequency exponent, respectively [37,38]. I represents the current intensity. While shot noise from barrier tunneling exhibits α=1, flicker noise dominated by the adsorption and desorption of gas molecules exhibits quadratic current dependence, α=2 [39]. The fitting value of α of the DNWs is 1.94, which is very close to the value of flicker noise from adsorption and desorption. Furthermore, if the fluctuation originates mainly from the adsorption and desorption of gas molecules, the value of ξ is between 0.5 and 1.5, while if the fluctuation originates from the generation–recombination of charge carriers in the mid-gap states or at surface states, the value of ξ is ≥1.5 [40]. ξ of the DNWs ranges from 0.92 to 1.32, indicating that when the current density is below 100 mA/cm^2^, in N_2_ or an air atmosphere, the adsorption and desorption of molecules are the main mechanism of noise generation, which is consistent with the experimental phenomenon described above. Specifically, ion bombardment induces adsorption, desorption, and migration of residual gas molecules, resulting in current fluctuations in the form of steps and spikes.

The turn-on fields and current fluctuation rates under different test current densities for various field emission structures of different materials are summarized in Table 8 [41,42,43,44,45,46,47]. The DNWs achieved a current fluctuation rate as low as 0.70% at 0.1 mA/cm^2^, with fluctuation rates below those of other field emission cathodes across the 0.1–10 mA/cm^2^ range, demonstrating stable electron emission performance over large current ranges.

## 5. Conclusions

In this paper, the FE properties and current stability of the DNW cathodes under different vacuum degrees, current densities, and atmospheres were systematically investigated. The experiments demonstrate that the FE properties and stability of the DNWs are improved with a decrease in pressure. At low current densities below 10 mA/cm^2^, the DNWs operate in field-induced adsorption mode. As the current density increases, the FE properties and stability gradually degrade. In the high-emission current region, the DNWs enter the current-induced desorption mode, and the FE properties and stability are improved. N_2_ exhibits superior FE performance and stability relative to compressed air and ambient air, attributed to its higher adsorption energy, minimal work function increase upon adsorption, and reduced ionization cross-section. The FE noise power spectrum of the DNWs was obtained through FFT, presenting the form of $P_{n}\left(f\right)=A_{0}\left(I^{\alpha }/f^{\xi }\right)$. The fitting value of the current exponent α is 1.94, and the frequency exponent *ξ* ranges from 0.92 to 1.32, indicating that the noise mechanism of the DNWs arises from the change in the surface work function induced by the adsorption and desorption of the residual gas. The results show that the DNWs demonstrate a low turn-on field of 1.65 V/μm and a high current density of 265.38 mA/cm^2^, attributable to the nanoscale curvature radius, large aspect ratio, and NEA. Under the test conditions of 1.2 × 10^−4^ Pa and 0.1 mA/cm^2^, a current fluctuation rate as low as 0.70% is achieved. The current fluctuation rate of the DNWs is less than 5% under all tested conditions, demonstrating excellent resistance to ion bombardment of residual gas, tolerance to pressure variations, and environmental stability in different gas environments. DNWs have great potential in applications such as vacuum microelectronics, precision sensors, and compact X-ray sources. Future work will focus on in-situ thermal processing, nanostructure optimization of DNWs, and composite material development to further suppress gas adsorption and enhance the current stability.

## Figures and Tables

**Table 1:** FE properties of the DNWs under different vacuum degrees.

Vacuum Degree (Pa)	1.2 × 10^−4^		1.0 × 10^−3^		1.3 × 10^−2^	
$E_{t}$ (V/μm)	1.84		2.23		3.50	
$J_{0}$ (mA/cm^2^)@100 V	249.04		212.50		200.08	
Slope of F-N curve	−16.70	−20.95	−20.84	−26.09	−23.38	−28.11
Rate of ∆∅ (%)		0		15.75		21.66

**Table 2:** FE current stability of the DNWs under different vacuum degrees with an applied voltage of 20 V.

Vacuum Degree (Pa)	1.2 × 10^−4^	1.0 × 10^−3^	1.3 × 10^−2^
$\bar{I}$ (A)	6.79 × 10^−5^	6.03 × 10^−5^	4.43 × 10^−5^
δ (A)	9.34 × 10^−7^	1.50 × 10^−6^	2.11 × 10^−6^
$\delta /\bar{I}$ (%)	1.37	2.48	4.76
ξ	0.95	1.24	1.09

**Table 3:** FE properties of the DNWs at different current densities.

Test Current Density (mA/cm^2^)	0.1		1		10		50		100	
$E_{t}$ (V/μm)	1.75		1.94		2.14		2.04		1.84	
$J_{0}$ (mA/cm^2^)@100 V	242.31		241.35		232.69		236.54		239.42	
Slope of F-N curve	−18.28	−19.69	−18.91	−21.82	−18.15	−24.08	−17.42	−23.84	−21.88	−20.08
Rate of ∆∅ (%)		0		7.08		14.37		13.59		1.32

**Table 4:** FE current stability of the DNWs at different current densities.

Test Current Density (mA/cm^2^)	0.1	1	10	50	100
Applied voltage (V)	3.3	9.8	27.0	44.6	61.3
$\bar{I}$ (A)	1.01 × 10^−6^	9.99 × 10^−6^	1.00 × 10^−4^	5.00 × 10^−4^	1.00 × 10^−3^
δ (A)	7.04 × 10^−9^	1.07 × 10^−7^	1.48 × 10^−6^	8.04 × 10^−6^	1.30 × 10^−5^
$\delta /\bar{I}$ (%)	0.70	1.07	1.48	1.61	1.30
ξ	0.92	1.12	1.30	1.28	1.30

**Table 5:** FE properties of the DNWs under different atmospheres.

Atmosphere	N_2_		Compressed Air		Air	
$E_{t}$ (V/μm)	1.65		1.86		2.13	
$J_{0}$ (mA/cm^2^)@100 V	265.38		248.08		233.65	
Slope of F-N curve	−19.51	−17.14	−17.59	−20.17	−18.98	−21.69
Rate of ∆∅ (%)		0		11.45		16.99

**Table 6:** Adsorption energy on a hydrogen-terminated diamond surface, work function after adsorption, and ionization cross-section at 20 eV incident electron energy of different gases [30,31,32,33,34,35,36].

Gas	$E_{ad}$ (eV)	∅ (eV)	*σ* (10^−16^ cm^2^)@20 eV
O_2_	−5.01	6.5	0.293
N_2_	+2.45	5.5	0.218
H_2_O	−0.26	4.9	0.457

**Table 7:** FE current stability of the DNWs under different atmospheres with an applied voltage of 20 V.

Atmosphere	N_2_	Compressed Air	Air
$\bar{I}$ (A)	7.23 × 10^−5^	6.79 × 10^−5^	4.80 × 10^−5^
δ (A)	9.01 × 10^−7^	9.34 × 10^−7^	9.22 × 10^−7^
$\delta /\bar{I}$ (%)	1.25	1.37	1.92
ξ	1.05	1.32	1.20

**Table 8:** Current fluctuation rate of field emission structures with different materials [41,42,43,44,45,46,47].

Field Emission Structure	$E_{t}$ (V/μm)	Test Current Density (mA/cm^2^)	Current Fluctuation Rate (%)
4H-SiC nanowires [41]	0.95	0.7	2.1
N-SiC nanoneedles [42]	1.22	1.1	6.5
SiC nanowires [43]	1.50	0.7	3.8
B-SiC nanoneedles [44]	1.92	0.5	6.5
AlN nanorods [45]	8.8	10.3	2
Si-AlN nanoneedles [46]	1.8	10	5
P-SiC nanoparticles [47]	1.03	2.65	3.0
DNWs	1.36	0.1	0.70
	1.94	1	1.07
	2.14	10	1.48

## Data Availability

The data that support the findings of this study are available within the article.
